# ALBI-sarcopenia score as a predictor of treatment outcomes in hepatocellular carcinoma

**DOI:** 10.1038/s41598-025-97295-7

**Published:** 2025-04-26

**Authors:** Maha Elsabaawy, Hanaa Badran, Amr Ragab, Rasha Abdelhafiz, Madiha Nageeb, Reham Ashour

**Affiliations:** 1 Hepatology and Gastroenterology Department, National Liver Institute, Menoufia University, Shebeen El-Koom, Egypt; 2 Radiodiagnosis and Interventional Radiology Department, National Liver Institute, Menoufia University, Shebeen El-Koom, Egypt

**Keywords:** ALBI, Sarcopenia, MELD, HCC, ALBI-Sarcopenia, Gastroenterology, Oncology

## Abstract

The recently developed ALBI-Sarcopenia score has demonstrated effectiveness in predicting mortality in hepatocellular carcinoma (HCC), emerging as a crucial factor in guiding treatment decisions. To assess the utility of the ALBI-Sarcopenia score in predicting the success of HCC treatment. A prospective study involving 262 liver cirrhosis with HCC patients were assigned to various treatment strategies according to Barcelona clinics of liver disease (BCLC) classification. Patients were followed up for 12 months reporting laboratory data, sarcopenia, ALBI-Sarcopenia score, and outcomes. Sarcopenia was prevalent in 43.1% (48.35% males and 31.25% females, *P* = 0.042). Most patients were HCV-positive (88.9%) and classified as CTP A (55.7%) or BCLC B (54.2%). Over the study period, TACE was the most administered treatment (41.2% at baseline), followed by a progressive shift toward best supportive care as disease severity increased. Complete response rates declined from 31.7% at 1 month to 21.4% at 12 months, while progressive disease rates increased from 21.8 to 37.8% over the same period. At 12 months, the ALBI-Sarcopenia score demonstrated the highest predictive accuracy for treatment response (AUC:0.69, *p* = 0.001), outperforming both the ALBI (AUC: 0.631, *p* = 0.001) and MELD (AUC:0.623, *p* = 0.003) scores. Logistic regression identified ALBI-Sarcopenia as a significant independent predictor of response at 1 month (OR:1.25, 95% CI:0.881–1.971, *p* = 0.002) and 12 months (OR:2.189, 95% CI:0.992–4.937, *p* = 0.001). The ALBI-Sarcopenia score is a robust predictor of treatment outcomes in HCC, offering superior prognostic accuracy compared to traditional scoring systems, and enhancing patient stratification for optimized treatment planning.

## Introduction

Hepatocellular Carcinoma (HCC) poses a significant global health challenge, ranking as the sixth most common cancer and the fourth leading cause of cancer-related mortality worldwide^1^. The complex interplay of genetic, environmental, and viral factors contributes to the development and progression of HCC, making it imperative to explore innovative approaches for prognostication and therapeutic stratification^2^. In this context, the Albi-Sarcopenia Score emerges as a promising and multifaceted tool that not only incorporates hepatic function assessment but also integrates the evaluation of skeletal muscle mass, shedding light on the intricate relationship between liver health and systemic body composition^3^.

The Albi-Sarcopenia Score amalgamates the Albumin-Bilirubin (ALBI) grade, a reliable indicator of liver function, with sarcopenia, a condition characterized by the loss of skeletal muscle mass and function^4^. While the ALBI grade has gained recognition for its simplicity and accuracy in predicting outcomes in HCC^5^, the incorporation of sarcopenia into the assessment introduces a novel dimension, considering the significant impact of muscle depletion on patient survival and treatment response^6^.

The intricate relationship between liver disease and skeletal muscle mass has been increasingly recognized as a crucial determinant of prognosis in various malignancies, and HCC is no exception^7^. The dual assessment provided by the Albi-Sarcopenia Score presents a holistic perspective, capturing both hepatic and extrahepatic factors that influence the trajectory of HCC^3^.

The significance of predicting treatment response in HCC cannot be overstated, given the evolving landscape of therapeutic modalities, ranging from surgical interventions to locoregional therapies and systemic treatments^8^. Identifying patients who are more likely to benefit from specific therapeutic approaches is paramount for optimizing outcomes and resource utilization. In the current clinical study, we aim to provide a comprehensive overview of the prognostic value of the Albi-Sarcopenia Score in diverse treatment settings for HCC.

## Patients and methods

### Study design and patient selection

This prospective observational study aimed to investigate the predictive value of the Albi-Sarcopenia Score in determining treatment response among patients diagnosed with HCC. It was conducted at the HCC multidisciplinary clinic, National Liver Institute, Menoufia University, Egypt, from May 2022 to May 2023.

This study was conducted in accordance with the Declaration of Helsinki and was approved by the Ethics Committee of the National Liver Institute, Menoufia University, Egypt (Approval No.: NLI IRB 00014014/FWA00034015, issued in February 2022). Written informed consent was obtained from all participants prior to study enrollment.

Consecutive patients with histologically confirmed HCC or those meeting non-invasive diagnostic criteria, as per the most recent BCLC guidelines for treatment allocation and prognostication^9^.

**Inclusion criteria** were based on well-defined parameters, with all patients having a confirmed diagnosis of cirrhosis-related HCC, classified according to the Barcelona Clinic Liver Cancer (BCLC) staging system. Patients without cirrhosis were not included, as cirrhosis is a well-established risk factor for HCC. All HCV-positive patients had undergone antiviral treatment as part of Egypt’s national HCV eradication program, making HCV viremia an irrelevant factor in our cohort.

**Exclusion criteria** included patients with acute illnesses, severe infections, or conditions that could significantly alter muscle mass and sarcopenia assessment. Patients with incomplete follow-up data or missing key clinical variables were also excluded, ensuring data integrity.

### Data collection

Baseline demographic and clinical data were collected for each participant, including age, gender, body mass index (BMI), aetiology of liver disease, presence of cirrhosis, and Barcelona Clinic Liver Cancer (BCLC) stage at the time of HCC diagnosis. Laboratory parameters, such as serum albumin, bilirubin, alanine aminotransferase (ALT), aspartate aminotransferase (AST), and alpha-fetoprotein (AFP), were measured using standardized assays.

The baseline was defined as the first evaluation encounter at our dedicated HCC multidisciplinary clinic. This initial assessment was performed prior to the institution of any new therapeutic interventions, ensuring that the baseline data (including laboratory tests, imaging for sarcopenia assessment, and prognostic scores) reflected the untreated status of the disease. Patients were enrolled consecutively at this first visit.

Sarcopenia was assessed through cross-sectional imaging, typically utilizing computed tomography (CT) scans or magnetic resonance imaging (MRI). Skeletal muscle mass was determined by measuring the cross-sectional area of the muscles at the level of the third lumbar vertebra (L3). The skeletal muscle index (SMI) was calculated as the cross-sectional muscle area normalized for height (cm^2^/m^2^). Sarcopenia was defined as skeletal muscle index ≤ 50 cm^2^/m^2^ for males and ≤ 39 cm^2^/m^2^ for females^4^.

The Albi-Sarcopenia Score was derived by combining the ALBI grade, calculated based on serum albumin and bilirubin levels, with the presence of sarcopenia. ALBI grades 1, 2, and 3 were assigned corresponding scores of 1, 2, and 3, respectively^3^. Sarcopenia was dichotomously categorized as present or absent, with a score of 1 assigned for the presence of sarcopenia and 0 for its absence. The final Albi-Sarcopenia Score was the sum of these two components, ranging from 0 to 4.

**Calculations**:

MELD = 10 * ((0.957 * ln (Creatinine)) + (0.378 * ln (Bilirubin)) + (1.12 * ln (INR) + 6.43^10^.

MELD-sarcopenia = MELD + (10.35 * Sarcopenia)^11^.

ALBI = − 0.085 × (albumin g/L) + 0.66 × log (bilirubin µmol/L)^12^.

**The newly developed score (Albi-Sarcopenia)**:

If sarcopenia = 9.

For ALBI = 7 for each unit increase in ALBI.

ALBI-Sarcopenia score = 9*sarcopenia + 7*ALBI^3^.

Treatment Modalities and Response Assessment: Patients received standard-of-care treatments based on the BCLC staging system, including surgical resection, liver transplantation, locoregional therapies (such as trans-arterial chemoembolization), and systemic therapies (such as sorafenib or immunotherapy)^13^. Treatment response was assessed using radiological imaging at predetermined intervals according to the respective treatment modalities.

**Fig. 1:**
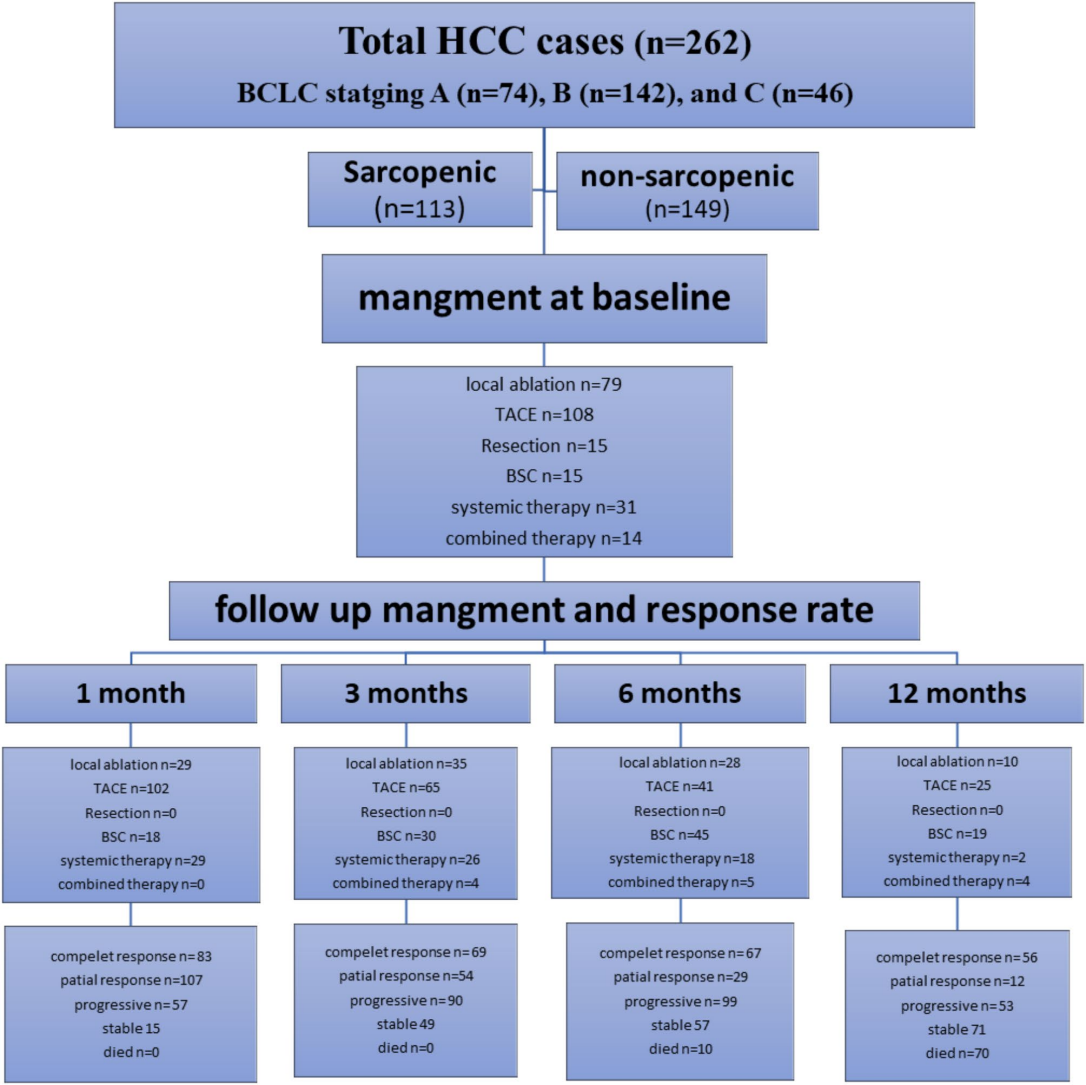
Flowchart of patients enrollment and follow-up.

Objective response was categorized based on the Response Evaluation Criteria in Solid Tumours (RECIST) guidelines, considering complete response (CR), partial response (PR), stable disease (SD), and progressive disease (PD). Treatment response was dichotomized into responders (CR/PR) and non-responders (SD/PD) for analysis^14^ (Fig. 1).

### Statistical analysis

The statistical analysis for this study was conducted using IBM SPSS Statistics for Windows, Version 20.0 (IBM Corp., Armonk, NY, USA). URL: https://www.ibm.com/products/spss-statistics.

Descriptive statistics were used to summarize patient characteristics. The association between the Albi-Sarcopenia Score and treatment response was assessed using logistic regression analysis, adjusting for relevant covariates. All statistical analyses were performed using a predetermined significance level of 0.05, and data were analysed using statistical software (SPSS). Subgroup analyses were conducted to explore potential variations in the predictive value of the Albi-Sarcopenia Score across different treatment modalities and disease stages.

## Results

Sarcopenia was present in 43.1% of patients, with a significantly higher prevalence in males (48.35%) compared to females (31.25%) (*P* = 0.042). Most patients had HCV-related liver disease (88.9%), were classified as Child-Pugh A (55.7%), and belonged to BCLC stage B (54.2%). Patients with sarcopenia had significantly larger tumor sizes (median: 4.0 cm vs. 3.4 cm in non-sarcopenic patients, *P* = 0.015) and a higher median number of focal lesions (2 vs. 2, *P* = 0.003). Obesity was more prevalent in non-sarcopenic patients (74.1% vs. 25.9%, *P* < 0.001), and the BMI was significantly lower in sarcopenic patients (mean 23.25 ± 2.14 kg/m^2^ vs. 25.15 ± 2.45 kg/m^2^, *P* < 0.001) (Table 1).

The most frequently administered treatment was TACE (41.2%), reflecting its role in intermediate-stage HCC. Curative treatments were less common: radiofrequency ablation (RFA) was performed in 15.3%, surgical resection in 5.7%, and microwave ablation in 9.2%. A progressive shift toward best supportive care (BSC) was observed (5.7%), particularly in BCLC C patients (32.6%). Systemic therapy was used in 11.8% of patients, with a majority receiving sorafenib (67.4%) (Table 2).

Complete response (CR) rates declined from 31.7% at 1 month to 21.4% at 12 months, while progressive disease (PD) increased from 21.8 to 37.8%, highlighting the challenge of achieving sustained tumor control. Partial response (PR) was observed in 40.8% at 1 month, declining to 29.2% at 12 months. Stable disease (SD) remained around 37% at 12 months (Table 3).

### Prognostic value of ALBI, MELD, MELD-sarcopenia, and ALBI-sarcopenia in predicting treatment response)

At 1 month, the ALBI-Sarcopenia score had the highest predictive accuracy (AUC: 0.628, *P* = 0.001), outperforming ALBI (AUC: 0.586, *P* = 0.033) and MELD (AUC: 0.609, *P* = 0.006). At 12 months, the ALBI-Sarcopenia score maintained the highest predictive accuracy (AUC: 0.69, *P* = 0.001), significantly outperforming the MELD score (AUC: 0.623, *P* = 0.003) (Table 4).

### Types of treatment among sarcopenic and non-sarcopenic groups

Sarcopenic patients were significantly less likely to receive curative treatments compared to non-sarcopenic patients: At baseline, only 24.8% of sarcopenic patients were eligible for curative treatment vs. 44.3% of non-sarcopenic patients.

**Fig. 2:**
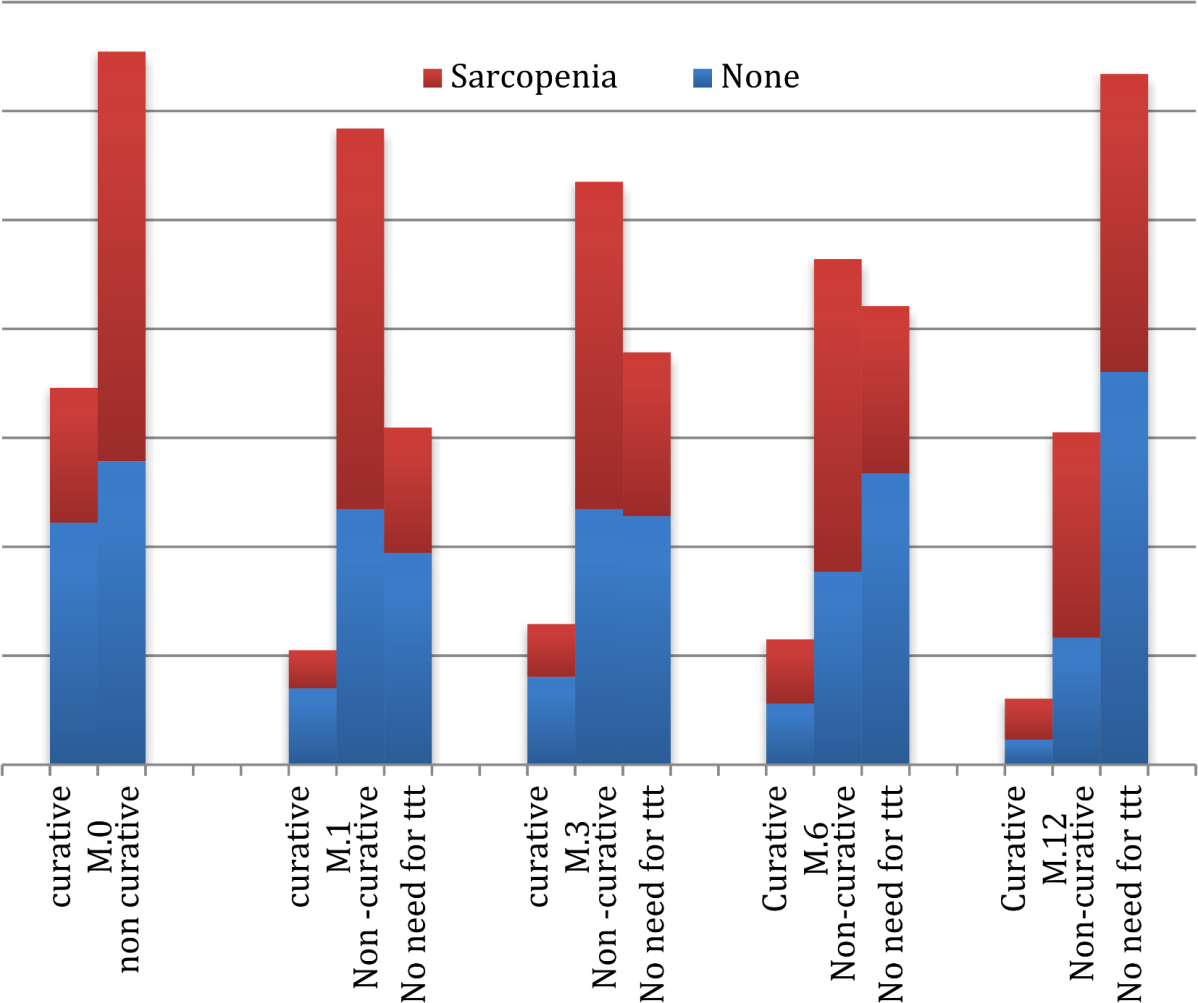
Types of treatment among sarcopenic and non sarcopenic groups. Curative Treatment for HCC: Liver transplantation, Surgical resection (liver resection), Ablation therapies (Radiofrequency ablation, Microwave ablation), Liver-directed therapies (e.g., after TACE). Non-Curative Treatment for HCC: Transarterial chemoembolization (TACE), Systemic therapy (Targeted therapy, Immunotherapy), Radiation therapy. No need for treatment: Palliative care.

By 1 month, 7% of sarcopenic patients remained eligible for curative treatment vs. 14% of non-sarcopenic patients. By 12 months, the proportion had further declined to 7.5% in sarcopenic patients vs. 4.6% in non-sarcopenic patients. The proportion of patients achieving complete response (CR) was significantly lower in sarcopenic patients: After 1 month: 23.0% vs. 38.9% (*P* = 0.017). After 3 months: 30.1% vs. 45.6% (*P* = 0.017). After 6 months: 30.7% vs. 53.5% (*P* = 0.017). After 12 months: 54.7% vs. 72.1% (*P* = 0.017) (Fig. 2).

### Effect of different grades of sarcopenia on HCC treatment response

At 1 month, patients with higher grades of sarcopenia had poorer responses: Complete response (CR) was highest in Grade 1 sarcopenia (67.5%) and lowest in Grade 3 (1.2%). Progressive disease (PD) was significantly more frequent in Grade 3 sarcopenia (14.1%) compared to Grade 0 (10.6%). Stable disease (SD) was most common in Grade 1 (46.7%) but least in Grade 3 (13.3%) (*P* = 0.106).

At 3 months, response patterns continued to diverge based on sarcopenia severity: CR remained highest in Grade 1 sarcopenia (68.1%) and lowest in Grade 3 (1.4%). PD increased in patients with Grade 2 and 3 sarcopenia (46.6% and 7.8%, respectively), indicating a worsening disease trajectory. Stable disease was more frequently observed in Grade 1 patients (67.4%) compared to Grade 3 (6.2%) (*P* = 0.11). At 6 months, the effect of sarcopenia on treatment response became more pronounced: CR was highest in Grade 1 (73.1%) and absent in Grade 3 (0%) (*P* = 0.210). PD rates surged in patients with Grade 3 sarcopenia (61.2%), further highlighting the adverse impact of muscle depletion on treatment efficacy.

At 12 months, sarcopenia had a strong impact on treatment outcomes: CR was highest in Grade 1 patients (76.8%) but drastically lower in Grade 3 (1.8%) (*P* = 0.122). PD remained most common in Grade 2 (60.4%) and Grade 3 (7.5%) patients. Stable disease was significantly higher in Grade 1 (81.7%) compared to Grade 3 (0%) (Table 5).

### Overall survival among sarcopenic and non-sarcopenic patients

Sarcopenic patients had significantly shorter median survival (10.09 months) compared to non-sarcopenic patients (11.72 months, *P* = 0.001). The 1-year mortality rate was significantly higher in sarcopenic patients (51.3%) than non-sarcopenic patients (10.1%), *P* = 0.012 (Table 6).

### Predictive accuracy of scoring systems for treatment response

The ALBI-Sarcopenia score had the highest AUC at 1 month (0.628, *P* = 0.001) and 12 months (0.69, *P* = 0.001), confirming its superior predictive performance over traditional models. The MELD-Sarcopenia score was also predictive at 1 month (AUC: 0.609, *P* = 0.006), supporting the integration of sarcopenia into prognostic models (Table 7).

### Univariate and multivariate logistic regression analysis of response predictors at 1 and 12 months)

BCLC stage A was strongly associated with a better response at 1 month (OR: 6.13, *P* < 0.001) but lost significance at 12 months (*P* = 0.236). Sarcopenia status remained a significant predictor at both 1 month (OR: 0.94, *P* = 0.008) and 12 months (OR: 0.79, *P* = 0.001). The ALBI-Sarcopenia score showed the strongest association with response at 1 month (OR: 0.93, *P* = 0.002) and 12 months (OR: 0.78, *P* = 0.001) (Tables 8 and 9).

**Table 1:** Comparison between none sarcopenia and sarcopenia.

	None (*n* = 149)	Sarcopenia (*n* = 113)	Test of Sig.	*P*
Gender				
Male	96 (52.7%)	86 (47.3%)	χ^2^ = 4.131	0.042^*^
Female	53 (66.3%)	27 (33.8%)		
Number of Fls				
Mean ± SD.	1.71 ± 0.87	2.07 ± 1.05	U = 6740.5^*^	0.003^*^
Median (Min–Max.)	2 (1–6)	2 (1–6)		
Size of Fls				
Mean ± SD.	3.88 ± 1.79	5.0 ± 3.24	U = 6945.5^*^	0.015^*^
Median (Min–Max.)	3.40 (1.30–11.0)	4.0 (1.70–15.0)		
Co-morbidity				
Diabetes mellitus	47 (58.8%)	33 (41.3%)	χ^2^ = 0.166	0.684
Obesity	86 (74.1%)	30 (25.9%)	χ^2^ = 25.305^*^	< 0.001^*^
BMI (kg/m^2^)				
Mean ± SD.	25.15 ± 2.45	23.25 ± 2.14	t = 6.564^*^	< 0.001^*^
Median (Min–Max.)	25.0 (21.0–33.0)	23.0 (19.0–29.0)		
Nutritional status	29 (70.7%)	12 (29.3%)	χ^2^ = 3.808	0.051
Performance status				
PS0	105 (66.9%)	52 (33.1%)	χ^2^ = 16.899^*^	< 0.001^*^
PS1	34 (44.7%)	42 (55.3%)		
PS2	10 (34.5%)	19 (65.5%)		

SD: Standard deviation, U: Mann Whitney test, χ^2^: Chi square test, p: p value for comparing between None Sarcopenia and Sarcopenia, *: Statistically significant at *p* ≤  0.05.

**Table 2:** Relation between performance status and BCLC (*n* = 262).

	Performance status			χ^2^	*p*
	PS0 (*n* = 157)	PS1 (*n* = 76)	PS2 (*n* = 29)		
BCLC					
BCLC A	65 (41.4%)	9 (11.8%)	0 (0.0%)	61.092^*^	<0.001^*^
BCLC B	80 (51.0%)	49 (64.5%)	13 (44.8%)		
BCLC C	12 (7.6%)	18 (23.7%)	16 (55.2%)		

χ^2^: **Chi square test**, p: p value for Relation between Performance Status and BCLC, *: Statistically significant at *p* ≤ 0.05.

**Table 3:** Descriptive analysis of the studied cases according to delta change in different parameters.

	Baseline (*n* = 262)	1 month (*n* = 262)	3 month (*n* = 262)	6 month (*n* = 250)	12 month (*n* = 191)	Change (*n* = 191)	*P* value
Albumin						Decrease	
Min.–Max.	2.40–4.60	2.50–34.0	2.30–4.70	1.90–4.50	2.0–5.0	– 1.30 to 2.0	
Mean ± SD.	3.50–0.44	3.60–2.25	3.29–0.47	3.28–0.47	3.48–0.56	0.11 ± 0.54	0.03*
Median (IQR)	3.50 (3.10–3.90)	3.40 (3.0–3.80)	3.20 (2.90–3.70)	3.20 (2.90–3.70)	3.0 (3.0–4.0)	0.10 (-0.20–0.45)	
PLT						Decrease	
Min.–Max.	25.0–336.0	30.0–252.0	34.0–290.0	34.0–289.0	39.0–319.0	– 75.0 to 273.0	
Mean ± SD.	105.36–53.70	98.13–38.44	94.95–37.73	93.03–33.19	98.69–37.59	11.48 ± 47.45	0.3
Median (IQR)	96.50(68.0–123.0)	90.0(74.0–114.0)	89.0(69.0–113.0)	88.0(76.0–105.0)	90.0(76.0–114.0)	0.0 (– 13.50 to 25.0)	
AFP						Increase	
Min.–Max.	3.0–18356.0	2.60–11562.0	2.90–109376.0	3.20–91587.0	2.10–9000.0	– 1089.6 to 8914.0	
Mean ± SD.	205.2–1160.6	203.6–937.5	917.7–8623.4	593.6–5890.9	122.7–680.7	29.99 ± 687.8	0.4
Median (IQR)	41.0(15.0–117.0)	35.65(15.0–125.0)	34.8(15.30–122.4)	31.5(15.40–137.6)	19.2(10.70–50.70)	– 2.60(– 37.9 to 12.0)	
CS Psoas						Decrease	
Min.–Max.	27.0–74.0	23.50–72.50	25.40–71.50	25.40–70.50	24.70–115.0	– 71.00 to 17.50	
Mean ± SD.	48.14–8.95	47.13–8.91	45.90–8.72	45.46–8.67	48.26–10.06	2.09 ± 7.84	0.001*
Median (IQR)	47.0(42.0–54.0)	46.20(41.0–53.0)	45.30(40.0–52.30)	44.6(39.60–51.90)	47.5(41.80–53.40)	2.50 (-0.20–5.0)	

**Table 4:** Distribution of the patients studied according to treatments at baseline in each BCLC grade.

Treatment at baseline	Total (*n* = 262)	BCLCA (*n* = 74)	BCLCB (*n* = 142)	BCLCC (*n* = 46)
RFA	40 (15.3%)	28 (37.8%)	12 (8.5%)	0 (0.0%)
PEI	15 (5.7%)	4 (5.4%)	11 (7.7%)	0 (0.0%)
Microwave	24 (9.2%)	17 (23.0%)	7 (4.9%)	0 (0.0%)
TACE	108 (41.2%)	10 (13.5%)	98 (69.0%)	0 (0.0%)
Resection	15 (5.7%)	15 (20.3%)	0 (0.0%)	0 (0.0%)
BSC	15 (5.7%)	0 (0.0%)	0 (0.0%)	15 (32.6%)
Sorafenib	31 (11.8%)	0 (0.0%)	0 (0.0%)	31 (67.4%)
Sorafenib combined	1 (0.4%)	0 (0.0%)	1 (0.7%)	0 (0.0%)
Combined intervention	13 (5%)	0 (0%)	13 (9.2%)	0 (0%)

BSC: best supportive care, TACE: trans-arterial chemoembolization, RFA: radiofrequency ablation, PEI: percutaneous ethanol injection.

**Table 5:** Effect of different grades of sarcopenia on response of HCC to treatment:

		Sarcopenia				Total	P value
		Grade O.	Grade 1	Grade 2	Grade 3		
		25 (9.56%)	148 (56.5%)	71 (27.1%)	18 (6.9%)	N (%)	
1st month	Stable	1 (6.6%)	7 (46.7%)	5 (33.3%)	2 (13.3%)	15 (5.7)%	*P* = 0.106
	Progressive	6 (10.57%)	28 (49.12%)	15 (26.3%)	8 (14.1%)	57 (21.8)%	
	Partial response	12 (11.21%)	60 (56%)	27 (25.2%)	7 (65.4%)	107 (40.8)%	
	Complete response	6 (7.2%)	56 (67.46%)	24 (29%)	1 (1.2%)	83 (31.7)%	

		Grade O.	Grade 1	Grade 2	Grade 3	Total	
3rd month		13 (5%)	147 (56%)	89 (34%)	13 (5%)	262	*P* = 0.11
	Stable	4 (8.2%)	33 (67.4%)	9 (18.4%)	3 (6.2%)	49 (18.7%)	
	Progressive	4(4.4%)	37 (41.1%)	42 (46.6%)	7 (7.8%)	90 (34.4%)	
	Partial response	0 (0%)	30 (55.5%)	22 (40.8%)	2 (3.7%)	54 (20.6%)	
	Complete response	5 (7.2%)	47 (68.1%)	16 (23.2%)	1 (1.4%)	69 (26.3%)	

		Grade O.	Grade 1	Grade 2	Grade 3	Total	
6th month		13 (5.1%)	153 (60.7%)	72 (29.3%)	11 (4.4%)	250	*P* = 0.210
	Stable	8 (14%)	40 (70.1%	6 (10.5%)	3 (5.3%)	57 (22.6%)	
	Progressive	4 (4.1%)	52 (53.1%	36 (36.7%)	6 (61.2%)	98 (39.3%)	
	Partial response	1 (3.3%)	12 (40%)	15 (50%)	2 (6.6%)	30 (11.5%)	
	Complete response	1 (1.5%)	49 (73.1%)	17 (25.4%)	0 (0%)	67 (26.6%)	

		Grade O.	Grade 1	Grade 2	Grade 3	Total	
12th month		9 (4.6%)	122 (64%)	53 (28.6%)	5 (2.6%)	189	*P* = 0.122
	Stable	7 (9.8%)	58 (81.7%)	6 (8.5%)	0 (0%)	71 (37.0%)	
	Progressive	0 (0%)	17 (32.1%)	32 (60.4%)	4 (7.5%)	53 (27.6%)	
	Partial response	0 (0%)	5 (41.6%)	7 (58.3%	0 (0%)	12 (6.3%)	
	Complete response	2 (3.6%)	43 (76.8%	10 (17.8%)	1 (1.8%)	56 (29.2%)	

**Table 6:** Overall survival among sarcopenic and non-sarcopenic patients.

Survival		Non-sarcopenia	Sarcopenia	*P*
Survival time	Mean (months)	11.72	10.09	0.001
	95% C.I	11.57–11.88	9.65–10.53	
1 year-mortality rate	Alive	134 (89.9%)	55 (48.7%)	0.012
	Dead	15 (10.1%)	58 51.3%)	

HCC: Hepatocellular carcinoma, CI: confidence intervals, P: p value.

**Table 7:** Prognostic value of ALBI, MELD, MELD sarcopenia, and ALBI-Sarcopenia score to predict response to treatment.

		AUC	*P*	95% C. I	Cut off	Sensitivity	Specificity	PPV	NPV
At 1 month (*n* = 190 vs. 72)	ALBI	0.586	0.033^*^	0.507–0.664	≤ – 2.01^#^	62.11	56.94	79.2	36.3
	MELD Sarcopenia	0.609	0.006^*^	0.533–0.685	≤ 18^#^	67.37	52.78	79.0	38.0
	ALBI-Sarcopenia	0.628	0.001^*^	0.552–0.704	≤ – 10.18	62.63	58.33	79.9	37.2
	Sarcopenia	0.0606	0.001*	0.508–0.644		62.11	56.94	79.19	36.28
	AFP	0.701	< 0.001^*^	0.629–0.773	≤ 56.5	67.37	65.28	83.7	43.1

AUC: Area Under a Curve, p-value: Probability value, CI: Confidence Intervals, NPV: Negative predictive value, PPV: Positive predictive value, *: Statistically significant at *p* ≤ 0.05, #Cut off was choose according to Youden index.

**Table 8:** Univariate logistic regression analysis of response predictors at 1, 12 months.

	At 1 month		At 12 months	
	*P*	OR (LL–UL 95%C.I)	*P*	OR (LL–UL 95%C.I)
ALBI	0.044^*^	0.514 (0.269–0.983)	0.001^*^	0.319 (0.160–0.637)
MELD	0.339	0.949 (0.853–1.056)	0.002^*^	0.829 (0.738–0.932)
MELD-Sarcopenia	0.008^*^	0.940 (0.898–0.984)	0.979	0.999 (0.955–1.046)
ALBI-Sarcopenia	0.002^*^	0.925 (0.881–0.971)	0.001^*^	2.189 (0.992–4.937)

OR: Odd`s ratio, C.I: Confidence interval, LL: Lower limit, UL: Upper Limit, *: Statistically significant at *p* ≤ 0.05.

**Table 9:** Multivariate logistic regression analysis of predictors of treatment response at 1 and 12 months.

Variable	1-Month Response OR (95% CI)	*P*-value	12-Month Response OR (95% CI)	*P*-value
BCLC stage (A vs. B/C)	6.13 (2.53–14.88)	< 0.001*	1.43 (0.79–2.60)	0.236
ALBI Sscore	0.51 (0.27–0.98)	0.044*	0.32 (0.16–0.64)	0.001*
MELD score	0.95 (0.85–1.06)	0.339	0.83 (0.74–0.93)	0.002*
Sarcopenia status	0.94 (0.90–0.98)	0.008*	0.79 (0.68–0.92)	0.001*
ALBI-Sarcopenia score	0.93 (0.88–0.97)	0.002*	0.78 (0.67–0.91)	0.001*
MELD-Sarcopenia score	0.94 (0.89–0.98)	0.008*	0.81 (0.70–0.94)	0.003*

*OR: Odds Ratio; CI: Confidence Interval; *P* ≤ 0.05 indicates statistical significance.

## Discussion

The exploration of predictors of HCC treatment response represents a significant stride towards personalized and precision medicine in the realm of HCC^15^. The integration of the ALBI score with sarcopenia in a new validated score had gained acceptance in predicting mortality in HCC patients^3^. The performance of this new score in predicting treatment responses was analysed in the current study.

In the current study, the demographic data reveals a male population (69.5%) with a mean age of 59.61 ± 8.09 years. Half of the patients were classified as sarcopenic, emphasizing the relevance of considering muscle mass in HCC patients undergoing treatment as an essential element in determining the course and treatment responses. Badran et al. had signified this role, stressing the linkage between sarcopenia and deterioration of liver condition and progression of HCC^16^.

Regarding the gender distribution among patients with sarcopenia, we noticed that sarcopenia was more prevalent in males than females (76.1% vs. 23.9%), with a statistically significant difference between patients with sarcopenia and those without (*P* = 0.042). In concordance with our findings, Montano-Loza and his colleagues, reported that sarcopenia was more common in males compared to females (50% vs. 18%, *P =* 0.001)^11^. In a similar vein, a study investigated sex differences in sarcopenia prevalence among 1,105 older adults in rural eastern China. Sarcopenia was more prevalent in women (21.7%) than men (12.9%). After adjusting for confounders, women had a 1.49-fold higher likelihood of sarcopenia than men (PR = 1.49, 95% CI [1.01–2.26], *P* = 0.055), suggesting potential sex-specific risk factors^17^. This high prevalence in males than in females might be attributed to that, women usually store the abundance of fat and generate their energy more preferentially from fat stores than from skeletal muscle stores^18^.

Notably, 38 out of the 262 patients (approximately 14.5%) were diagnosed through a dedicated HCC surveillance program. These surveillance-detected patients generally presented with earlier-stage disease, lower tumor burden, and better performance status compared to those who were diagnosed following symptom onset. This observation is consistent with previous studies that highlight the benefits of regular HCC surveillance in high-risk populations, leading to earlier detection and improved prognostic outcomes^9^. The inclusion of this subgroup reinforces the critical role of systematic surveillance in facilitating timely and potentially curative interventions.

In evaluating the distribution of patients based on treatment responses, it was evident that the majority experienced either complete or partial responses at 1, 3, 6, and 12 months, indicating that most patients were diagnosed with HCC at earlier stages permitting better performance of the different therapeutic strategies. However, a considerable proportion faced disease progression or stability, indicating the clinical heterogeneity of HCC and the challenges associated with achieving sustained treatment success.

ALBI, MELD, and MELD-Sarcopenia performed well in the prognostication of HCC treatment responses in the current study. Remarkably, the ALBI-Sarcopenia score demonstrates the highest AUC and statistically significant predictive capability, making it the best overall predictor among the scores at both 1 month and 12 months. ALBI-Sarcopenia and MELD Sarcopenia show statistically significant AUC values, indicating moderate predictive ability for treatment response at 1 month. Montano-Loza et al. were the first to mention the applicability of MELD Sarcopenia to evaluate patients with HCC and predict mortality in behaviour better than both MELD and sarcopenia alone^11^. Elsebaey et al. had assessed the performance of this combination in predicting mortality in treated HCC patients with robust evidence of efficacy and even superiority over other known scores^19^. Ultimately, Elsabaawy et al. reported the excellence of ALBI-Sarcopenia over ALBI, MELD, and MELD Sarcopenia in predicting mortality in HCC patients^3^.

ALBI score was assigned as an essential element in predicting HCC treatment responses in many studies with different treatment strategies, either curative or palliative^20–23^. Remarkedly, it was relied on in the randomized controlled trial of KEYNOTE-240 evaluating the response to HCC immune therapy^24^.

Likewise, the MELD score is crucial and widely accepted in assessing the prognosis or severity of liver disease in HCC patients^25,26^. In contrast to the accepted role of the MELD score in predicting outcomes for HCC patients, the present study’s findings indicated that the MELD score alone may not be statistically significant in predicting early responses to treatment (at 1 month). However, it still holds a predictive value in forecasting outcomes at later stages (12 months). These results might be explained by the dynamic nature of HCC and Treatment Response, which may evolve, and early changes might not be accurately captured by a single assessment, such as the MELD score at 1 month. Additionally, the type and duration of treatment could impact how well the MELD score predicts outcomes. Some treatments might delay liver function, and the MELD score might not fully capture these changes early during treatment.

The prognostic value of ALBI, MELD, and sarcopenia in portraying HCC treatment strategies was assessed in many studies with proven efficacy^27–29^.

Notably, the superiority of ALBI over the MELD score had been robustly reported for simplicity, specificity to liver functions only, better survival discriminative ability, and applicability to all BCLC stages^30,31^. This was the rationale for endorsing sarcopenia with ALBI in a new score, testing its ability to foretell the outcome of HCC patients.

In the current study, the performance of BCLC in foretelling HCC outcomes was robustly evidenced. Added to its importance to all HCC patients as an important tool in the allocation of patients in all treatment options either curative or palliative, BCLC might be a good option instead of resorting to the costly ALBI-Sarcopenia score. This critical query about the importance of ALBI-Sarcopenia in this context might be resolved by the fact that most HCC needs CT for proper diagnosis which is the diagnostic tool of sarcopenia at the same time. Remarkably, it is important to survey sarcopenia in HCC patients for treatment which might improve both outcome and quality of life in this critically ill patient^31^.

While the study sheds light on the promising predictive capability of the ALBI-Sarcopenia score, there are some limitations to be considered. The relatively small sample size and the single-center nature of the study may impact generalizability. Further research is warranted to validate these findings in larger, multicenter cohorts.

## Conclusion

The ALBI-Sarcopenia score’s enhanced predictive accuracy is crucial for tailoring treatment strategies in HCC patients. By incorporating muscle mass assessment, this score accounts for a critical aspect of patient health that traditional liver function scores may overlook. This comprehensive approach allows for better stratification of patients, ensuring that those at higher risk of poor outcomes receive more intensive monitoring and tailored therapeutic interventions.

## Data Availability

“The data used to support the findings of this study are available from the corresponding author upon request”.

## Abbreviations

HCC: Hepatocellular carcinoma
ALBI: Albumin bilirubin
MELD: A model for end-stage liver disease
BCLC: Barcelona clinic liver cancer
TACE: Trans-arterial chemoembolization
RFA: Radiofrequency ablation
CTP: Child-Pugh classification

## References

[1] Llovet, J. M. et al. Hepatocellular carcinoma. *Nat. Rev. Dis. Primers* **7**(1), 6 (2021). 33479224 10.1038/s41572-020-00240-3 PMC13537433

[2] Boyce, W. T., Sokolowski, M. B. & Robinson, G. E. Genes and environments, development and time. *Proc. Natl. Acad. Sci. USA*. **117**(38), 23235–23241 (2020). 32967067 10.1073/pnas.2016710117 PMC7519332

[3] Elsabaawy, M. et al. Appraisal of a newly developed ALBI-sarcopenia score as a prognostic marker in patients with hepatocellular carcinoma. *Eur. J. Gastroenterol. Hepatol.* **36**(7), 924–928. 10.1097/MEG.0000000000002776 (2024). 38625821 10.1097/MEG.0000000000002776

[4] Cruz-Jentoft, A. J. et al. Sarcopenia: revised European consensus on definition and diagnosis. *Age Ageing**48*(1), 16–31. 10.1093/ageing/afy169 (2019). 10.1093/ageing/afy169 PMC6322506 30312372

[5] Deng, M., Ng, S. W. Y., Cheung, S. T. & Chong, C. C. N. Clinical application of Albumin-Bilirubin (ALBI) score: the current status. *Surgeon* **18**(3), 178–186 (2020). 31601470 10.1016/j.surge.2019.09.002

[6] Zhang, X. M. et al. Sarcopenia as a predictor of mortality among the critically ill in an intensive care unit: a systematic review and meta-analysis. *BMC Geriatr.* **21**(1), 339 (2021). 34078275 10.1186/s12877-021-02276-w PMC8173733

[7] Uojima, H. et al. Skeletal muscle mass influences tolerability and prognosis in hepatocellular carcinoma patients treated with lenvatinib. *Liver Cancer* **9**(2), 193–206 (2020). 10.1159/000504604 PMC7206580 32399433

[8] Tsujita, Y. et al. Evaluation and prediction of treatment response for hepatocellular carcinoma. *Magn. Reson. Med. Sci.* **22**(2), 209–220 (2023). 36792205 10.2463/mrms.rev.2022-0118 PMC10086401

[9] Reig, M. et al. BCLC strategy for prognosis prediction and treatment recommendation: the 2022 update. *J. Hepatol.* **76**(3), 681–693 (2022). 34801630 10.1016/j.jhep.2021.11.018 PMC8866082

[10] Wiesner, R. et al. Model for end-stage liver disease (MELD) and allocation of donor livers. *Gastroenterology* **124**, 91–96 (2003). 12512033 10.1053/gast.2003.50016

[11] Montano-Loza, A. J. et al. Inclusion of sarcopenia within MELD (MELD-Sarcopenia) and the prediction of mortality in patients with cirrhosis. *Clin. Translational Gastroenterol.* **6**, e102 (2015). 10.1038/ctg.2015.31 PMC4816259 26181291

[12] Johnson, P. J. et al. Assessment of liver function in patients with hepatocellular carcinoma: a new evidence-based approach-the ALBI grade. *J. Clin. Oncol.* **33**, 550–558 (2015). 25512453 10.1200/JCO.2014.57.9151 PMC4322258

[13] Makary, M. S., Khandpur, U., Cloyd, J. M., Mumtaz, K. & Dowell, J. D. Locoregional therapy approaches for hepatocellular carcinoma: recent advances and management strategies. *Cancers (Basel)*. **12**(7), 1914. 10.3390/cancers12071914 (2020). 32679897 10.3390/cancers12071914 PMC7409274

[14] Nishino, M. et al. New response evaluation criteria in solid tumors (RECIST) guidelines for advanced non-small cell lung cancer: comparison with original RECIST and impact on assessment of tumor response to targeted therapy. *AJR Am. J. Roentgenol.* **195** (3), W221–W228 (2010). 20729419 10.2214/AJR.09.3928 PMC3130298

[15] Galun, D. et al. Precision medicine for hepatocellular carcinoma: clinical perspective. *J. Pers. Med.* **12**(2), 149 (2022). 35207638 10.3390/jpm12020149 PMC8879044

[16] Badran, H. et al. Baseline sarcopenia is associated with a lack of response to therapy, liver decompensation, and high mortality in hepatocellular carcinoma patients. *Asian Pac. J. Cancer Prev.* **21**(11), 3285–3290 (2020). 33247686 10.31557/APJCP.2020.21.11.3285 PMC8033124

[17] Yang, Y. et al. Prevalence of sarcopenia was higher in women than in men: a cross-sectional study from a rural area in Eastern China. *PeerJ* **10**, e13678. 10.7717/peerj.13678 (2022). 35935249 10.7717/peerj.13678 PMC9354735

[18] Ou, M. Y. et al. Adipose tissue aging: mechanisms and therapeutic implications. *Cell. Death Dis.* **13**, 300. 10.1038/s41419-022-04752-6 (2022). 35379822 10.1038/s41419-022-04752-6 PMC8980023

[19] Alsebaey, A. et al. MELD-Sarcopenia is better than ALBI and MELD score in patients with hepatocellular carcinoma awaiting liver transplantation. *Asian Pac. J. Cancer Prev.* **22**(7), 2005–2009 (2021). 34319020 10.31557/APJCP.2021.22.7.2005 PMC8607083

[20] Fagenson, A. M., Gleeson, E. M., Pitt, H. A. & Lau, K. N. Albumin-bilirubin score vs model for end-stage liver disease in predicting post-hepatectomy outcomes. *J. Am. Coll. Surg.* **230**, 637–645 (2020). 31954813 10.1016/j.jamcollsurg.2019.12.007

[21] Waked, I. et al. Transarterial chemoembolization of hepatocellular carcinoma: impact of liver function and vascular invasion. *Br. J. Cancer* **116**, 448–454 (2017). 28125820 10.1038/bjc.2016.423 PMC5318968

[22] Vogel, A. et al. ALBI score and outcomes in patients with hepatocellular carcinoma: *post hoc* analysis of the randomized controlled trial KEYNOTE-240. *Ther. Adv. Med. Oncol.* **13**, 17588359211039928. 10.1177/17588359211039928 (2021). 34616489 10.1177/17588359211039928 PMC8488519

[23] Cheng, Y. T. et al. IS. MELD score is the better predictor for 30-day mortality in patients with ruptured hepatocellular carcinoma treated by trans-arterial embolization. *Am. J. Cancer Res.* **11**(7), 3726–3734 (2021). 34354871 PMC8332870

[24] Lau, T. & Ahmad, J. Clinical applications of the model for End-Stage liver disease (MELD) in hepatic medicine. *Hepat. Med.* **5**, 1–10 (2013). 24696621 10.2147/HMER.S9049 PMC3953735

[25] Marasco, G. et al. Clinical impact of sarcopenia assessment in patients with hepatocellular carcinoma undergoing treatments. *J. Gastroenterol.* **55**(10), 927–943 (2020). 32748172 10.1007/s00535-020-01711-w PMC7519899

[26] Guerrini, G. P. et al. Value of HCC-MELD score in patients with hepatocellular carcinoma undergoing liver transplantation. *Prog Transpl.* **28**(1), 63–69 (2018). 10.1177/1526924817746686 29251164

[27] Liu, P. H. et al. ALBI and PALBI grade predict survival for HCC across treatment modalities and BCLC stages in the MELD era. *J. Gastroenterol. Hepatol.* **32**(4), 879–886 (2017). 27696519 10.1111/jgh.13608

[28] Pinato, J. et al. The ALBI grade provides objective hepatic reserve estimation across each BCLC stage of hepatocellular carcinoma. *J. Hepatol.* **70**(4), 696–703 (2019). 10.1016/j.jhep.2016.09.008 27677714

[29] Hiraoka, A., Michitaka, K., Kumada, T. & Kudo, M. ALBI score as a novel tool in staging and treatment planning for hepatocellular carcinoma: advantage of ALBI grade for universal assessment of hepatic function. *Liver Cancer* **6**, 377–379 (2017). 29234641 10.1159/000481212 PMC5704723

[30] Demirtas, C. O., D’Alessio, A., Rimassa, L., Sharma, R. & Pinato, D. J. ALBI grade: evidence for an improved model for liver functional estimation in patients with hepatocellular carcinoma. *JHEP Rep.* **3**(5), 100347 (2021). 34505035 10.1016/j.jhepr.2021.100347 PMC8411239

[31] Perisetti, A. et al. Sarcopenia in hepatocellular carcinoma: current knowledge and future directions. *World J. Gastroenterol.* **28**(4), 432–448 (2022). 35125828 10.3748/wjg.v28.i4.432 PMC8790553

